# Family planning use and associated factors among pastoralist community of afar region, eastern Ethiopia

**DOI:** 10.1186/s12905-016-0321-7

**Published:** 2016-07-18

**Authors:** Mussie Alemayehu, Hailemariam Lemma, Kidan Abrha, Yohannes Adama, Girmatsion Fisseha, Henock Yebyo, Ejigu Gebeye, Kassahun Negash, Jemal Yousuf, Tigist Fantu, Tesfay Gebregzabher, Araya Abrha Medhanyie

**Affiliations:** School of Public Health, Mekelle University, P.O.Box 1871, Mekelle, Ethiopia; AMREF Health Africa in Ethiopia, Addis Ababa, Ethiopia; Institute of Public Health, University of Gondar, Gondar, Ethiopia; Mekelle University College of Health Sciences School of Public Health, P.O. Box 1871, Mek’ele, Ethiopia

**Keywords:** Afar, Family planning, Utilization, Ethiopia, Pastoralist community

## Abstract

**Background:**

Ethiopia is the second most populous country in Africa with a total fertility rate (TFR) of 4.8 children per a woman and contraceptive prevalence rate (CPR) of 29 %. The overall prevalence of modern family planning in a pastoralist community, like Afar region, is low (9.1 %). This study aimed to assess family planning utilization and associated factors among married women of Afar region, Eastern Ethiopia.

**Methods:**

A community-based cross-sectional study was conducted from January 10-28, 2013 among 602 women. Multistage sampling technique was used to select the study participants. Descriptive and multiple variable logistic regression analyses were done to isolate independent predictors on utilization of family planning using SPSS 20.

**Results:**

The overall prevalence of family planning utilization in Afar region was 8.5 % (6.2–10.7). Majority of the women (92.2 %) had used injectable. The most common reasons mentioned in the non-use of family planning methods were religion-related (85.3 %), desire to have more children (75.3 %), and husband's objection (70.1 %). Women who had a positive attitude towards family planning utilization (AOR = 4.7, 95 % CI: 2.1, 10.3), owning radio (AOR = 1.8, 95 % CI: 1.02, 4.18), and literate (AOR = 4.4, 95 % CI: 1.80, 11.08) were more likely to use family planning methods as compared to their counterparts. The increase of monthly income was also associated with the likelihood of family planning methods utilization. The odds of using family planning methods were higher among those with monthly income of $27–$55.5 (AOR = 2. 0, 95 % CI: 1.9, 4.7) and > $55 (AOR = 4. 6, 95 % CI: 1.23–17.19) as compared to women with the lowest category of monthly income ($27 and less).

**Conclusion:**

The low coverage of family planning in the region could be due to the influence of husband, religious and clan leader**.** Attitude of women towards family planning methods, possession of radio, monthly income, and educational status could influence family planning utilization.

## Background

Family planning (FP) has been identified by the World Health Organization (WHO) as one of the essential reproductive health interventions needed to achieve safe motherhood by reducing maternal and child mortality [1]. Effective FP could save the lives of 100,000–200,000 women and more than 1 million infants [2]. Despite the current global increment in the prevalence of modern contraceptive use, many developing countries are still suffering from the consequences of high unmet needs for modern contraceptives [2].

More than 350 million couples worldwide has limited or no access to effective and affordable FP [2]. Sub-Saharan African (SSA) countries have the lowest contraceptive use (17 %) and the highest unmet need (24 %) for modern FP among married women of childbearing age [3]. As more than half of the people in Africa are younger than 25 years old, unmet need is expected to increase as these individuals enter their reproductive years [4]. In this region the current prevalence of contraceptive use would have to triple by 2015 in order to satisfy existing demand for FP [5, 6].

Ethiopia is the second most populous country in Africa with an estimated population of 91.73 million based on the projection of the 2007 census. The national contraceptive prevalence rate (CPR) and total fertility rate (TFR) is reported as 29 % and 4.8 respectively [7]. However, Ethiopia has articulated in its population policy and health sector development program (HSDP IV) to achieve a TFR of 4.0 and a CPR of 66 % by 2015 [8]. Almost one quarter of the country’s currently married women (25 %) have an unmet need for FP [7]. This will require quite concentrated effort to increase the country’s CPR and shift the mixed method with greater emphasis on modern contraceptive. In Ethiopia, the pastoralist community represents about 11 % of the total population and 52 % of the country’s geographic area. The Afar region is one of the biggest pastoralist communities in the country. The overall health status of the Afar population is poor. According to the Ethiopia Demography and Health Survey (EDHS) report in 2011, the overall use of modern FP in the region was (9.1 %); where pill use was 0.3 %, injectables 7.6 %, and implants2%. Yet, none of the women in Afar region had used female sterilization, Intrauterine Contraceptive Device (IUCD), and male condoms. In addition to the physical inaccessibility of the residential areas and poor infrastructure, the nomadic feature of the community makes access to health facility worse. The people are underserved in all forms of healthcare, and its health status is not well documented [9].

Providing effective modern FP services are one method of reducing maternal and child mortality in pastoral community settings. Understanding the factors that can influence FP use like attitude of women, educational status,monthly income and owing radio in pastoral community setting is needed to increase the uptake of services. Eventhough,the EDHS tries to identify the reason for using and not using of family planning, it was superficial and they didn’t dig out the root cause reason for not using of family planning. Therefore, this study aimed to point out the level of modern FP methods utilization and identify determinants of FP use among married women in the pastoral community of Afar region, Eastern Ethiopia. Findings of this study would provide evidence to FP program managers and service providers to design targeted approached. Besides, it could have a contrubtion in achiving growth transformation plan of the country and sustainble development goal.

## Methods

### Study area and period

The Afar region is one of the nine regions of Ethiopia, located in the eastern part of the country. Administratively, the region is divided into five zones, 32 districts and 404 sub-districts (the smallest administrative unit). The total population of the region is over 1.5 million, among which 43 % are female. Only 7.7 % of the population are literate and over 96 % of the population in the region lives on pastoral livestock [9]. A community-based cross-sectional study was conducted from January 10–28, 2013.

### Sample size and sampling technique

To determine the sample size, a single population proportion formula was used with the assumption of 9.1 % FP utilization in the region from the EDHS 2011 report [7]. In addition, with the assumption of 95 % confidence level and 3 % degree of precision and design effect of 1.6, to decrease the variability that might be introduced because of the sample complex, were assumed to compute the sample size. To compensate the expected non-responses, we added 10 % of the calculated sample size, amounting the total sample size to 621.

A multi-stage sampling technique was used to select the study participants. Since the living status, accessibility and culture of the community in Afar are somehow homogenous, we took only two zones out of the five. There were 11 districts within the two zones (6 districts from Zone 3 and 5 districts from Zone 5). We included two districts each from both zones randomly (*Awash, Burimudiatu, Dewe, and Kummame*). Down the hierarchy, five sub-districts from *Awash*, four from *Burmudita*, four from *Dewe* and three of *Kummame* districts were randomly selected. A total of 95,934 women of reproductive age population within 19,986 households lived in these sub-districts [9]. The total sample size was allocated based on probability proportion to size of the sub-districts that is based on the number of households. Within each sub-district, we selected women for interview at equal interval using systematic sampling techinque. Initially clockwise direction was used to select the first household from the center of the sub district. The sampling fraction (K^th^) was calculated for each “sub district” based on the available number of married women in the household. Finally since the sampling fraction was 5, every 5^th^ household with married women was included in the study. At last lottery method was used to start the first house hold from the 5^th^ interval.In a case where two or more eligible women were encountered in the same household, only one woman was considered in the study at random to avoid intra-class correlation.

### Measurement

A total of 10 items was used to measure the attitude question and it contains questionare like male involvement on the discussion and approval of family planning and religious perception on contraceptive use. For analysis items related to the attitude of the respondents towards family planning was grouped into three “strongly agree” and “agree”, as “agree”, “neutral” as “neutral” and, “disagree” and “strongly disagree” as “disagree”. Finally by adding the overall variables, the total score of family planning was done based on the mean value. Then, they were said to have Positive Attitude, if their score was above mean to the correct answers from attitude measuring family planning questions and having a negative attitude if they score mean and below the mean.

### Data collection

Structured and pre-tested questionnaire, guided by the interviewer, was used to collect the data. It was first prepared in English and then translated into Amharic and then translated back to English to check for consistency of the questions. We used Amharic language since there was difficult about writing the questions in Afarigna, the local language in the Afar region. However, the data collectors were all from Afar region and they were fluent in both Amharic and Afarigna**.** The questionnaire was adapted from different literatures and also considered the local situation of the study subjects [7, 10]. The questionnaire comprised data on socio-demographic and economic variables, reproductive history, knowledge, attitude and practice of FP. Eight female data collectors who can speak the local languages were employed in the data collection process. Two nurses were posted for supervision. Training was given to the data collectors and supervisors for two consecutive days for the purpose of the study, the contents of the questionnaire, particularly on issues related to the confidentiality of the responses, the rights of respondents, and the sampling procedure. Prior to the data collection, the questionnaire was pre-tested among 30 women in another distirct which was not incuded in the actual study.

### Data analysis

The raw data were entered into EPI data version 3.1 and analyzed using SPSS version 20 for Windows (SPSS Inc. version 20, Chicago, Illinois). Descriptive analyses were run to estimate the level of FP utilization and descriptions of women characteristics. The predictors of FP utilization were assessed using multiple logistic regression analysis. The effect size of predictors was estimated using adjusted Odds Ratio (OR) for the sample and 95 % CI of OR for the population effect sizes. A p-value of less than 0.05 was considered as statistically significant for all tests.

## Results

A total of 602 married women were participating in the study, which made the response rate to 97.8 %. The average age of the women was 27.7 years (SD = 2.6). Majority of the respondents were Afar in ethnicity (96.2 %), illiterate (89.7 %), and housewives (69.6 %). The vast majority of the women, (98.8 %), were Muslim. As expected, 98.5 % of the women used to live in rural areas. The average monthly income of a household was $23 (SD = 2.4). Out of the total participants, 239(39.7 %) and 25(4.2 %) had radio and television, respectively [Table 1].

**Table 1:** Socio-demographic characteristics of married women of pastoralist community of Afar region, 2013

Characteristics	Frequency	Percent
Age of married women (*n* = 602)		
15–19	45	7.5
20–24	136	22.6
25–29	169	28.1
30–34	125	20.8
35–39	84	14.0
> = 40	43	7.0
Religion (*n* = 602)		
Muslim	595	98.8
Others^a^	7	1.2
Ethnicity (*n* = 602)		
Afar	579	96.2
Others^b^	23	3.8
Residence (*n* = 602)		
Rural	593	98.5
Urban	9	1.5
Education status (*n* = 602)		
Not able to read and write	540	89.7
Primary school	37	6.1
Secondary school	21	3.5
College or University level	4	0.7
Occupational status (*n* = 602)		
Student	12	1.99
Employee	29	4.8
House wife	421	69.9
Merchant	20	3.32
Pastoralist	122	20.26
Own earn income (*n* = 602)		
Yes	51	8.5
No	551	91.5
House hold income (*n* = 602)		
<27 $	482	80.1
27–55.5 $	102	16.9
> 55.5 $	18	3.0

^a^Orthodox and protestant

^b^Tigre, Amhara, Argoba and Oromo

1$ = 18ETB

### Reproductive characteristics of women

The mean age at marriage was 15.72 years (SD = 2.7) and first birth was 16.9 years (SD = 2.8). On average, each women had 3.95 pregnancies (SD =2.6). Experience of at least one abortion was reported among 112 (18.6 %) of the respondents. Similarly, 32(5.3 %) of the women had a history of under five children death, of which 8(25 %) had two or more under five children deaths. Each woman had an average of 1.64 (SD = 0.03) under five children and more than half (53.1 %) had two or more under-five children. The average family size was 5.33 (SD =2.3); but 58.5 % had family size of four or more. Eight in ten of women wanted to have children for the future [Table 2].

**Table 2:** Reproductive history of married women of pastoralist community of Afar region, 2013

Characteristics	Number	Percent
Age at first marriage (*n* = 602)		
< 18	571	94.9
≥ 18	31	5.1
Age at first delivery (*n* = 602)		
< 18	483	78.6
≥ 18	129	21.4
Number of pregnancy (*n* = 602)		
≤ 2	209	34.7
≥ 3	393	65.3
Number of live born children (*n* = 602)		
≤ 2	234	38.9
≥ 3	368	61.1
Family size (*n* = 602)		
≤ 4	250	41.5
4 and above	352	58.5
Number of under five children (*n* = 474)		
One	222	46.9
Two and above	252	53.1
Face abortion (*n* = 602)		
Yes	112	18.6
No	490	81.4
Need future child (*n* = 602)		
Yes	511	84.9
No	91	15.1
Preference of sex (*n* = 511)		
Male	81	15.9
Female	129	25.2
As God/Allah give me	301	58.9
History of child death (*n* = 602)		
Yes	32	5.3
No	570	94.7
Number of child death (*n* = 32)		
One	24	75
Two and above	8	25

### Knowledge of married women about contraception

Three hundred seventy three (62 %) of the participants had ever heard about FP methods. The most widely known contraceptives were injectable 365(97.9 %), pill 333(89.3 %) and implanon 40(10.7 %). A small number of women had ever heard of male condoms 32(8.8 %), Jadelle 36(9.7 %), IUCD 20(5.4 %) and permanent contraceptive methods 18(5 %).

Health facilities were the main source of information for the respondents 150(40.2 %). Nearly eighty percent of the respondents knew the purpose of FP is for spacing. Surprisingly, all of the respondents did not know the purpose of FP as the prevention of STIs and HIV/AIDS. Majority of the women, (90 %), knew where to obtain contraceptives, of these more than three-fourth of the respondents mentioned that contraceptive methods are found in health centers. However, almost all (97.9–100 %) participants did not know that FP can be obtained from nongovernmental organizations (NGO), shops, private clinics and pharmacies [Table 3].

**Table 3:** Married women’s knowledge on family planning in pastoralist community of Afar region, 2013

Characteristics	Yes		No	
	No	%	No	%
Source of information (*n* = 373)				
Health facility	150	40.2	223	59.8
Family	33	8.8	340	91.2
Husband	18	4.8	355	95.2
Volunteers	56	15	317	85
Health extension workers	140	37.5	233	62.5
Friends	127	34	246	66
Nongovernmental organization	33	8.8	340	91.2
Private clinic	3	0.8	370	99.2
Mass media	36	9.7	337	90.3
Which mass media (*n* = 36)				
Radio	33	91.7	3	8.3
Television	6	16.7	30	83.3
Purpose of family planning (*n* = 373)				
Limiting of child	147	39.4	226	60.6
Child spacing	305	81.8	68	18.2
Prevent STI/HIV/AIDS	3	0.8	370	99.2
As treatment	6	1.6	367	98.4
Place where get family planning (*n* = 373)				
Health center	263	79.5	68	20.5
Health post	132	39.9	199	60.1
Hospitals	12	3.6	319	96.4
Private clinic	6	1.8	325	98.2
Pharmacy	7	2.1	324	97.9
NGO	0	0	331	100
Shop	1	0.3	330	99.7

### A**ttitudes of married women towards contraceptive utilization**

The overall attitude showed that only six in ten of the respondents had positive attitude towards FP utilization. Seventy percent of the women believed that having several children compensated for high child mortality and 62 % believed that having more children improves family income. In addition, 56.4 % considered that contraceptive use can cause infertility. Above half of the respondents believed that it is a sin to use FP with the perspectives of their religious faith. Nearly two-third of the participants thought that women who use FP methods would be abandoned by their husbands or have conflict with their husbands (73.8 %). The participants also believed that discussion among couples about FP use is mandatory (69.3 %), whereas a significant number (71.4 %) of the participants believed that the partner should approve their FP use. Although fewer, 13 % of the married women agreed that short-acting is more effective than long-acting contraceptive methods [Table 4].

**Table 4:** Attitude of married women towards utilization of family planning in pastoralist community of Afar region, 2013 (*n* = 602)

Characteristics	Agree		Neutral		Disagree	
	No	%	No	%	No	%
Having many children will improving the income of the family?	373	62	69	11.5	160	26
Contraceptive use causes infertility	338	56.4	164	27.2	100	16.6
High child mortality should be compensated by many births.	422	70.1	37	6.1	143	23.8
It is a sin to practice family planning methods with religious perspectives	467	77.6	85	14.1	50	8.3
Men should share the responsibility of family planning use	75	12.5	438	72.8	89	14.8
Women who practice family planning will be abandoned by their husbands	386	64.1	162	26.9	54	9
A couple that practice family planning methods will have conflict with their partner	444	73.8	107	17.8	51	8.5
Discussion among couple about family planning use is mandatory	417	69.3	123	20.4	62	10.3
My partner should approve family planning use	430	71.4	117	19.4	55	9.1
Short acting is more effective than long acting contraceptive	78	13	482	80	42	7

### Family planning utilization

Ever users of any contraceptives were 11.6 %; but, only 8.5 % (6.2–10.7) were using during the study period. Of the current users, nine in ten were using injectable. Nearly 84 % of the women reported that they were using FP methods for birth spacing. Majority of the women, 47 (92.2 %), received the contraceptives from governmental health institutions. Out of the current users, two-third (66.6 %) had received some forms of information (such as informed consent), but only 8(23.5 %) of them received information about side effects attributed to the FP methods, and 26(76.5 %) were informed about the availability of different choices. Of the total participants, only 66(11 %) of the women have ever discussed about FP methods with their husbands. Nearly 82(13.6 %) of the participants claimed to use FP methods in the future, most of them (88.2 %) prefer injectable over the others [Table 5].

**Table 5:** Different characteristics related to family planning utilization among married, reproductive age group women in Afar region, 2013

Characteristics	Number	Percent
Ever used FP (*n* = 602)		
Yes	70	11.6
No	532	88.4
Current use of FP (*n* = 602)		
Yes	51	8.5
No	551	91.5
Method currently using (*n* = 51)		
Injectables	47	92.2
pill	4	7.8
Purpose of FP method use (*n* = 51)		
Birth spacing	48	94.1
Birth limiting	1	2.0
Husband pressure	2	3.9
Source of methods (*n* = 51)		
Governmental health institutions	47	92.2
Voluntaries	4	7.8
Received Informed choice (*n* = 51)		
Yes	34	66.7
No	17	33.3
Information on Side effects FP methods (*n* = 34)		
Yes	8	23.5
No	26	76.5
Information on Available choices of FP(*n* = 34)		
Yes	26	76.5
No	8	23.5
Information on management of side effect (*n* = 34)		
Injectables	30	88.3
Pill	1	2.9
Implant 3 years	3	8.8
Discussed about FP with husband (*n* = 602)		
Yes	66	11
No	536	89
Future use of FP (*n* = 602)		
Yes	82	13.6
No	520	86.4

**Fig. 1:**
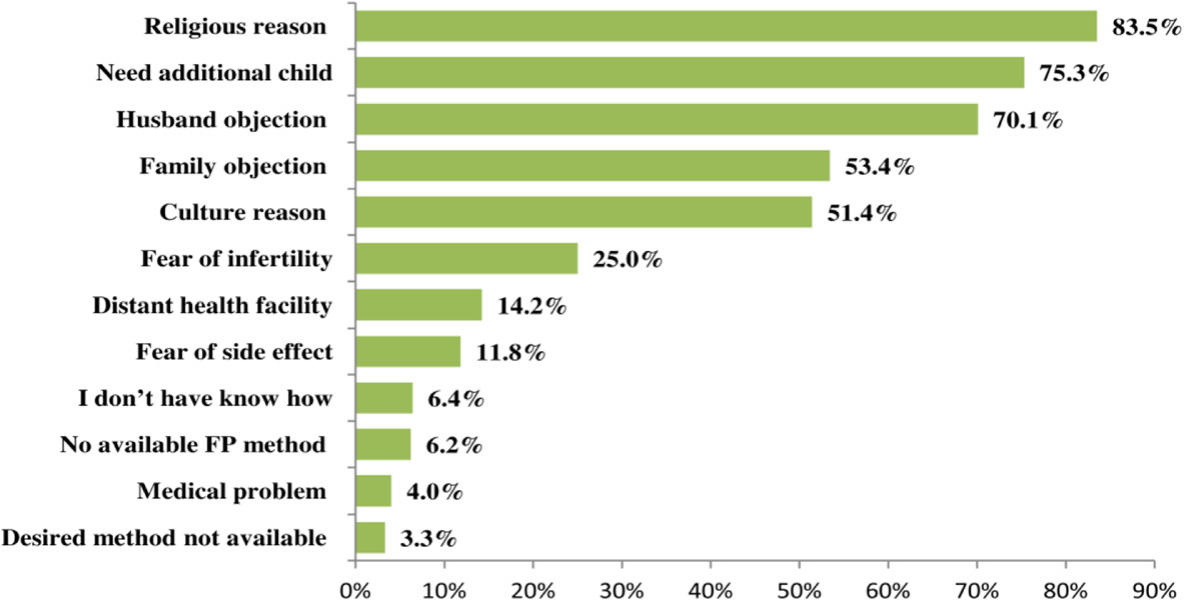
Reasons forwarded by married women for not using family planning methods at study period, Afar region, Ethiopia

Among the current users, 49(96.1 %) desired to continue use of family FP methods in the future. In contrast, 518 (94 %) of non-users will not have an interest to use any FP methods in the future. Generally, younger women had the more likelihood of using contraceptive; i.e., 26 (31 %) and 25(18.1 %) were using FP at the age of 15–24 and 25–39, respectively. But none of them hadn’t used among the women at the age of 40 or above. Among the current users, half of them (51 %) reported that decision to use FP methods was made by themselves without consulting their husbands. Whereas, the remaining either had discussed with their husbands (41.2 %) or their husband made the decision (7.8 %) on their wives’ FP utilization. Among the currently non-users of FP methods, 302(54.4 %), thought that they would use FP methods without the knowledge of their husband. Most of the women reported that the commonest reasons for not using FP methods were religion (85.3 %), desire to have more children (75.3 %), and husband's objection (70.1 %) [Figure 1].

### Factors assoacited with family planning utilization

Binary and multiple logistic regressions were fitted to identify the predictors of FP utilization. Attitude towards FP utilization, income catagories, and possession of radio, religion and educational status of women found to significantly predict FP utilization.

The odds of using FP methods was five times higher among women with positive attitude towards FP as compared to those with negative attitudes (AOR = 4.7, 95 % CI: 2.10, 10.3). Similarly, mothers who had radio had 2 times more odds to use FP as compared with those who did not have a radio (AOR = 1.8, 95 % CI: 1.02, 4.18). Mothers who were literate were more likely to use FP as compared with the illiterate (AOR = 4.4, 95 % CI: 1.80, 11.08). Monthly income was consistently and significantly associated with different income catagories. Mothers who had a monthly income of $27–$55.5 (AOR = 2.0, 95 % CI: 1.9, 4.7) and those with $55 or more had higher odds of using FP than the mothers with lowest income (<$27) (AOR = 4.6, 95 % CI: 1.2, 17.1) [Table 6].

**Table 6:** Determinant of family planning use among married women in pastoralist community Afar region, 2013

Characteristics	Use of FP		COR	AOR
	Yes	No		
	No (%)	No (%)		
Age category				
15–24	26(51)	155(28.1)	1	1
30–34	21(41.2)	279(50.6)	0.4(0.2,0.8)	0.5(0.2,1.13)
35–49	4(7.8)	117(21.2)	0.2(0.06,06)	0.4(0.1,1.92)
Number of pregnancy				
≤ 4	41(80.4)	327(59.3)	1	1
> 5	10(19.6)	224(40.7)	0.3(0.1,0.7)	1.1(0.4,3.1)
Attitude				
Negative	38(74.5)	351(63.7)	1	1
Positive	13(25.5)	200(36.3)	5.1(2.6,9.8)	4.7(2.1, 10.3)
Radio				
No	19(37.3)	344(62.4)	1	1
Yes	32(62.7)	207(32.6)	2.7(1.5,5.0)	2.0 (1.02,4.18)
Female education				
illiterate	24(47.1)	516(93.6)	1	1
literate	27(52.9)	35(6.4)	16.5(8.6,31.6)	4.4(1.8,11.08)
House hold income				
< 27 $	26(51)	456(82.8)	1	1
27–55.5 $	18(35.3)	84(15.2)	3.7(1.9,7.1)	2.0(1.9,4.7)
> 55.5 $	7(13.7)	11(2)	11.1(3.9,31.1)	4.6(1.23,17.19)

## Discussion

The low coverage of family planning in the region could be due to the influence of husband, religious and clan leader. Attitude of women towards family planning methods, possession of radio, monthly income, and educational status were associated with family planning utilization. Moreover, this study was conducted in a rural, remote, pastoralist community and married women where almost all (98.8 %) were Muslims and 90 % were unable to read and write.

Early marriage in populations where contraceptive prevalence is low is found to be highly correlated with high fertility. In this study, 95 % and 79 % of the women had their first marriage and first birth under the age of 18 years, respectively. Both are below the national figures, according the EDHS 2011, where the mean age for a first marriage and first birth were reported 16.5 years and 19.2 years, respectively [7]. The women age of their first marriage was against the Ethiopian Revised Family Code Proclamation, which declares that the minimum age of a first marriage is 18 for both sexes [8]. The early marriage and early time of child birth might have influence to have the maternal depletion syndrome and thus the negative affect maternal mortality and morbidity. This implies that the government of Ethiopia should give emphasis to increase the age at first marriage and birth to standard, particularly in pastoralist communities such as the Afar region.

The current study found that six in ten of the women have heard about FP methods. This was consistent with a study done in Pakistan-[11] - but lower than the figure in the EDHS [7]. Knowledge on FP is a prerequisite in obtaining access to and using suitable contraceptive methods, both in a timely and effective manner. Knowledge and attitude might influence FP utilization. In this sense, the government of Ethiopia and the respective stakeholders should render commensurate focus on creating awareness and knowledge of FP methods and utilization among mothers of the pastoralist communities. And this could be achived by strengthen the current structure of HEW program in Afar region. Thus,the HEW program brings a tremendous change in the other region of the country since they are working at the grassroot level which enables them to understand the community needs. For instance, rural women are more likey to use long acting contraceptive in Tigray region [12].

Information on source of contraceptive methods is important for policymakers, FP program managers, and implementers to adjust strategies of contraceptive delivering. Radio has emerged as one of the main sources of information for FP in many other studies, including the EDHS 2011 [7]. Similarly, the current study also found that one of the sources of information was radio. However, despite the fair possession (39.7 %), only 9.7 % of them heard about FP from radio. Moreover, eight in ten of the participants reported that health centers were sources of information for FP. This signifies that in addition to the need of expanding health centers, other strategies such as deployment and strengthening the capacity of health extension workers in the pastoralist communities is imperative to increase the access to information and FP methods. The health extension program through unreserved roles of health extension workers are being proved to improve the overall healthcare of the Ethiopian population in particular in the agrarian regions of the country.

One of the national reproductive health strategies of Ethiopia is to create acceptance and demand for FP, with special emphasis on population considered vulnerable by geographic dispersion, gender, and wealth to reduce unwanted pregnancies and enable individuals to achieve their desired family size [8]. However, in this study, a higher proportion (85 %) of the participants will want to have more children in the future, which was more than twofold as compared to EDHS 2011 [7]. This implies that the women in pastoralist regions of the country have low knowledge and attitude to use FP methods. This might be due to the influences from husband and religions leaders. As such, the government and stakeholders shall consider and work with religious leaders and involve husbands in FP awarenss creation and behavioural change programs in in the pastoralist communities of Ethiopia.

The proportion of ever users (11.6 %) and current users (8.5 %) of FP in this study were lower. This finding was below the national FP prevalence (29 %) and by far remote to the national target of CPR set at 66 % by 2015 [7, 8]. Similarly, the current figure is lower than the findings from Muslim communities of Jordan (53 %), Eastern Turkey (54.8 %), rural India (37.8–45.2 %) and Kelantan of Malaysia (31.8 %) [13–16]. Out of these findings from Muslim communities, we can understand that the religion by itself could not be an obstacle to FP utilization. Nevertheless, it might demand extensive efforts to create awareness and increase knowledge on the importance of FP through community workers, involvement of religious leaders, involvement husbands and other integrative endeavors to improve the FP utilization.

This study identifies a wide gap between knowledge (62 %) and practice (8.5 %) of FP among the participants. This can be explained by the poor attitude of the influential groups of the community in regards to FP use. Participants mentioned that, religion (85.3 %) and husbands’ objection (70.1 %) were the most common reasons for not using FP methods. This is also supported by findings of studies done in Pakistan and India in which religious unacceptability, desire for more children, fear of side effects, and illiteracy are important obstacles for non-use of FP methods [17, 18]. The huge gap existed between knowledge and practices should be bridged by changing the neagative attitude of the women towards using FP through varieties of strategies. One of which could be using the health extension workers, who are basically deemed to live with the communities, should read the contextual background and need of the community to intervene the low attitude of the women towards FP use.

Among the current users of FP, nine in ten of the respondents use injectable which correspond to the national average (72.4 %) and a study done in Arsi [7, 19]. Further, future intention of FP use among the women was injectable (88.2 %) as supported by the EDHS (96 %) [7]. This implies that a huge number of women in the study area as well as in the country as a whole prefer short-acting over the other contraceptive methods. However, short-acting contraceptive are less-effective, costly and needs repeated visits to health facilities. Availing method mix and addressing the barriers through decentralization of the service and training and deployment of health workers to all health facilities - [20] - could enhance the utilization of long-acting contraceptive methods.

The primary decision makers in regards to the use of FP among couples are one of the factors affecting the use of FP. Studies in Pakistan and Malaysia show that FP utilization was lower in women who often lacked decision making power on FP use. Objection from husbands was one of the major reasons for women refusing contraceptive use, especially among the Muslim women [16, 21]. Similarly, in this study a larger proportion of married women reported that they would be abandoned or would have a conflict with their husband if they use FP by their own decision.

Information about the intention of married women to use FP in the future is important for program managers and implementers. The majority (86.4 %) of study participants will not have an intention to use any FP methods in the future. This indicates that large effort is needed in creating awareness and bringing a positive attitude to increase FP utilization in the future by involving influential groups (such as husband and religious leaders) to influence the women to use FP since these groups of people have high acceptance by the community. Moreover, health providers are also expected to play major role in counseling women generally in use of FP methods, particularly in informed consent, FP options, availability of method mix and potential side effects [7].

Mothers who had positive attitude had higher odds of using FP. This implies that having a positive attitude of women towards contraceptive use is an important factor for promoting use of FP and creating a favorable environment on the acceptance of informed consent which is given by a health care provider.

In this study, mothers who were literate were more likely to use FP as compared with the illiterate and this was consistent with the finding of EDHS 2011 [7]. As expected, the increase in educational status increases the overall status of the women, including income, decision-making power, knowledge and attitude towards FP use and generally improves health seeking behavior. Although this takes a longer time, inter-collaboration with education sector is important to empower women through education to achieve most health indicators. Similarly, current use of any contraceptive method increases with increasing monthly income. This is also consistent with the report of EDHS 2011, which revealed that the contraceptive use increased from 13 % of women in the lowest quintile to 52 % of women in the highest quintile [7]. This implies that empowering women in income, education and decision making power are essential strategies to improve FP use and betterment of health status of the community.

This study might have limitations in that it didn’t test the influence of husbands on contraceptive use. Further, the community on which we studied is very conservative in their culture and religion. Besides, the data collector were responsible to interview the mother by translating the Amaharic language questionare to Afaraigna and this may result variability among the data collector. As such, the data we obtained from this community might lack slight validity. However, we believe that these limitations wouldn’t have much influence on the final estimates of each indicator since we employed female data collector from the local setting which understand the community context and strong supervion was made on daily basis.

## Conclusion

The low coverage of family planning in the region could be due to the influence of husband, religious and clan leader**.** In addition, the future intention of FP utilization is low. Early marriage and childbirth were common Attitude of women towards family planning methods, possession of radio, monthly income, and educational status could influence family planning utilization.

## Abbreviation

CPR, contraceptive prevalnce rate; EDHS, Ethiopian demographic health survey; FP, family planning; HSDP IV, health sector development program IV; IUCD, intra uterine contraceptive device; SPSS, stastical package for social sciences; SSA, Sub-Saharan Africa; WHO, World health organaization

## References

[1] United N. The Millennium Development Goals Report. New york: 2005. http://unstats.un.org/unsd/mi/pdf/mdg%20book.pdf. Accessed 10 January 2014.

[2] Jacob Stein R, Bakamjian L, Pile JM (2008). Threatened and still greatly needed family planning programs in Sub-Saharan Africa.

[3] WHO, UNICEF, UNFPA, World Bank (2007). Maternal mortality estimates in 2005.

[4] USAID, WHO (2008). Update on family planning in Sub-Saharan Africa. Repositioning family planning: guidelines for advocacy action. USAID, Africa’s Health in 2010.

[5] United State Agency for International Development (USAID. The case for long acting and permanent methods. FHI; 2007.

[6] UNFPA. Programme of Action of the International Conference on Population and Development. 2004. https://www.unfpa.org/sites/default/files/event-pdf/PoA_en.pdf.

[7] Central Statistical Agency [Ethiopia] and ORC Macro (2011). Ethiopia Demographic and Health Survey (EDHS) 2006.

[8] Federal democratic republic of Ethiopia. National Guideline for Family Planning Services in Ethiopia. Addis Abeba: 2011.

[9] CSA. Summary and statistical report of the 2007 population and housing census : population by age and sex composition. Addis Ababa: FDRE population census commission. December 2008. CSA.

[10] Alemayehu M, Belachew T, Tilahune T. Factors affecting utilization of long acting and permanent contraceptive among married women of reproductive age group in Mekelle Town. BMC Pregnancy Childbirth. 2012;12:6. http://www.ncbi.nlm.nih.gov/pmc/articles/PMC3297532/. Accessed 10 January 2014. 10.1186/1471-2393-12-6 PMC3297532 22280163

[11] Jabeen M, Gul F, Wazir F, Javed N. Knowledge, attitude and practices of contraception in women of reproductive age. Gomal Journal of Medical Sciences. 2011;9(2):223-9.

[12] Alemayehu M, Aster K, Alem D, Hailay G, Tesfalem H, Henock Y (2015). Rural women are more likely to use long acting contraceptive in Tigray region, Northern Ethiopia: a comparative community-based cross sectional study. BMC Womens Health.

[13] Paksima SM, Madanat HN, Hawks SR (2002). A contextual model for reproductive health education: fertility and family planning in Jordan. Promot Educ.

[14] Sahin HA, Sahin H (2003). Reasons for not using family planning methods in Eastern Turkey. Eur JContraceptReprod Health Care.

[15] Chandhick N, Dhillon BS, Kambo I (2003). Contraceptive knowledge, practices and utilization of services in the rural areas of India (an ICMR task force study). Indian J Med Sci.

[16] Norsa’adah B, Asmani AR, Noorliza MI, Tengku NT (2007). Acceptance and Knowledge of Family Planning Among Muslim Women in Rural Villages of Kelantan. JIMA.

[17] Sajid A, Malik S. Knowledge, Attitude and Practice of Contraception among Multiparous Women for Lady Aitchison Hospital, Lahore, Pakistan. ANNALS. 2010;16:4.

[18] Prachi R, Das GS, Ankur B, Shipa J, Binita K (2008). A study of knowledge, attitude and practice of family planning among the women of reproductive age group in Sikkim. J obstetGynecol India.

[19] Ko IS, You MA, Kim ES (2010). Family planning practice and related factors of married women in Ethiopia. Int Nurs Rev.

[20] United States Agency for International Development (USAID) (2008). Long-Acting and Permanent Methods of Contraception: Without them, a Country’s Development Will Be Low and Slow, the acquire project.

[21] Ying SL (1992). Determinants of fertility in Malaysia --how much do we know?. J Southeast Asian Stud.

